# Decreased Proportion of Cytomegalovirus Specific CD8 T-Cells but No Signs of General Immunosenescence in Alzheimer’s Disease

**DOI:** 10.1371/journal.pone.0077921

**Published:** 2013-10-14

**Authors:** Gabriel Westman, Anna-Karin Lidehall, Peetra Magnusson, Martin Ingelsson, Lena Kilander, Lars Lannfelt, Olle Korsgren, Britt-Marie Eriksson

**Affiliations:** 1 Department of Medical Sciences, Uppsala University, Uppsala, Sweden; 2 Department of Immunology, Genetics and Pathology, Uppsala University, Uppsala, Sweden; 3 Department of Public Health and Caring Sciences, Uppsala University, Uppsala, Sweden; Blood Systems Research Institute, United States of America

## Abstract

Cytomegalovirus (CMV) has been suggested as a contributing force behind the impaired immune responsiveness in the elderly, with decreased numbers of naïve T-cells and an increased proportion of effector T-cells. Immunological impairment is also implicated as a part of the pathogenesis in Alzheimer’s disease (AD). The aim of this study was to investigate whether AD patients present with a different CMV-specific CD8 immune profile compared to non-demented controls. Blood samples from 50 AD patients and 50 age-matched controls were analysed for HLA-type, CMV serostatus and systemic inflammatory biomarkers. Using multi-colour flow cytometry, lymphocytes from peripheral blood mononuclear cells were analysed for CMV-specific CD8 immunity with MHC-I tetramers A01, A02, A24, B07, B08 and B35 and further classified using CD27, CD28, CD45RA and CCR7 antibodies. Among CMV seropositive subjects, patients with AD had significantly lower proportions of CMV-specific CD8 T-cells compared to controls, 1.16 % vs. 4.13 % (p=0.0057). Regardless of dementia status, CMV seropositive subjects presented with a lower proportion of naïve CD8 cells and a higher proportion of effector CD8 cells compared to seronegative subjects. Interestingly, patients with AD showed a decreased proportion of CMV-specific CD8 cells but no difference in general CD8 differentiation.

## Introduction

Alzheimer’s disease (AD) is the most common form of dementing disorder and is characterised by a deterioration of cognitive and functional capacity. Due to ongoing demographic changes and the current lack of effective therapy, the socioeconomic burden of AD is estimated to increase globally in the years ahead [1]. 

Neuropathologically, the AD brain displays a progressive synaptic and neuronal loss together with extracellular plaques, mainly consisting of amyloid-β (Aβ), and intracellular neurofibrillary tangles of the microtubule-associated protein tau. According to the amyloid hypothesis, the pathogenesis is initiated by an increased production of Aβ followed by cytoskeletal changes and neuronal loss. Evidence for the primary role of Aβ has mainly been provided by the findings of disease-causing mutations in genes related to the generation of Aβ.

The pathology typically starts in the entorhinal cortex and other structures of the medial temporal lobe. However, with increased disease duration the pathology is extended in a hierarchical fashion to other cortical areas [2,3]. In addition to the main pathological changes, other features of the affected brain often include vascular alterations with deposition of Aβ in the vessel walls, especially in carriers of the Apolipoprotein (*APOE* ) ε4 allele [4], as well as various inflammatory reactions [5]. It is not completely understood how amyloid plaques, neurofibrillary degeneration, vascular alterations, inflammation and immune responses are related to each other and whether any infectious agents can influence the disease process.

The possible influence of viral infections on AD development has been investigated. For example, one study found that previous exposure to herpes simplex virus type 1 increased the risk of AD in carriers of the *APOE* ε4 allele [6] whereas later studies have failed to find such a correlation [7]. 

Whereas exposure to human cytomegalovirus (CMV) could influence the disease risk has not been extensively studied. CMV is a member of the betaherpesvirus group, causing a chronically persistent infection that in the immunocompetent adult rarely escapes immune surveillance, but can cause severe disease in patients with suppressed immune function [8,9]. Infection can occur in all stages of life, with a reported seroprevalence ranging from approximately 30 to 90% depending on age and ethnicity [10,11]. Recently, CMV has been shown to inflict a deep imprint in the host T-cell compartment that is characterised by an age-related oligoclonal expansion of differentiated CD8 (CD27-CD28-) cells and a corresponding decrease in proportion of naïve cells [12-15]. Also, the degree of differentiation in the CD4 compartments has been shown to correlate with levels of CMV IgG [16]. 

Alterations in systemic immunity have been shown to occur in the elderly, and the term *immunosenescence* is used to describe the age-related decline in capacity and regulatory balance of both innate and adaptive immune responses [17,18]. Dysregulation of immunoactive cells could also explain the progression of baseline systemic inflammation called *inflammaging*, which is considered a risk factor for several age-related diseases and where the role of CMV has been investigated [19]. Examples of clinically important immunosenescence include an impaired vaccine response that is especially pronounced in CMV seropositive patients [20,21] and an increased incidence of severe bacterial and viral infections in the elderly. In Swedish octogenarian and nonagenarian cohorts [22,23] a defined immune risk profile (IRP) consisting of a shift in CD4/CD8 ratio was associated with increased morbidity and mortality, but later studies conducted in different epidemiological settings have shown somewhat conflicting results [24].

Alzheimer’s disease has previously been associated with shifts in non CMV-specific CD4 as well as CD8 T-cell subsets [25-27]. Whether the CMV-specific CD8 immunity, shown to be of clinical importance for CMV disease in settings of more prominent immune deficiency [28], is affected in dementia patients has to our knowledge not previously been studied. Here, we have investigated if levels of CD8 T-cell CMV specificity and general CD8 differentiation differ in AD patients compared to non-demented (ND) controls.

## Methods

### Subjects

In the AD study group, a total of 51 patients were recruited from the Memory Clinic at the Department of Geriatrics in Uppsala University Hospital. All of them had recently undergone an extensive diagnostic work-up and received a clinical AD diagnosis in accordance with the NINCDS-ADRDA criteria [29] and DSM-IV criteria. Thus, all patients described a clinical picture of AD as well as a CT or MRI scan consistent with the diagnosis, i.e. with the absence of significant vascular abnormalities. Mini-mental State Examination (MMSE) scores were available from all but one and ranged from 10 to 27. In the ND control group, 52 age-matched subjects were recruited from a database of listed volunteers from the same geographical area as the AD group. Subjects in the ND control group had been recruited via local advertising and did not have any subjective cognitive impairment. Due to a change in diagnosis from AD to frontotemporal dementia, one participant in the AD group was later excluded and in the ND group two patients were excluded due to technical problems with sample handling prior to analysis, rendering a total of 50 AD patients and 50 ND controls. Dementia status was coded, allowing blinding during all laboratory work.

Informed consent was obtained in writing from all study participants, together with written consent from a close relative if there was any uncertainty on whether the subject was capable of providing informed consent him- or herself. The study was approved by the Regional Ethical Review Board in Uppsala, Sweden.

### Sampling and routine analyses

Blood samples were acquired by venipuncture, performed in accordance with standard clinical protocol. Routine analyses, including clinical chemistry and apolipoprotein E (*APOE*) genotyping, were made at the Department of Clinical Chemistry and Pharmacology, whereas HLA genotyping (PCR-SSO) was performed at the Department of Clinical Immunology and Transfusion Medicine, both accredited laboratories at Uppsala University Hospital.

PBMCs were isolated from blood samples collected in BD Vacutainer CPT™ Cell Preparation Tubes with sodium citrate. The tubes were kept at room temperature, before centrifugation and washing steps in accordance with the manufacturer’s protocol. A total of between 12-76 x 10^6^ cells were acquired per patient and frozen in batches of 5 x 10^6^ cells in medium consisting of 15% dimethyl sulfoxide (DMSO) and 85% foetal calf serum (FCS). All samples were stored in liquid nitrogen until analysis. The excess plasma from PBMC isolation was separately frozen in -20°C until analysed for CMV IgG (SIEMENS Enzygnost anti-CMV/IgG) at the Department of Clinical Microbiology at Uppsala University Hospital, and study participants were classified as either CMV seropositive or seronegative.

### Flow cytometry

For each study participant, one batch of 5 x 10^6^ cells were quickly thawed in a 37°C water bath and diluted in 40 ml cold wash buffer consisting of phosphate buffered saline (PBS) with 2.5% foetal bovine serum (FBS, Invitrogen) and 0.1% sodium azide. The suspension was centrifuged at 250 x *g* for 5 min and the supernatant was discarded. Next, the pellet was resuspended in 400 µl wash buffer and divided into four aliquots. In accordance with HLA typing results, titrated amounts of PE-labelled CMV-specific iTAg™Class 1 MHC tetramers (Beckman Coulter) were added to separate aliquots, if alleles matched one to four of the following: HLA-A*0101, HLA-A*0201, HLA-A*2402, HLA-B*0702, HLA- B*0801 or HLA-B*3501. If no tetramers matched, one sample was still analysed without any tetramers, rendering a total of 197 samples. Each sample was also concomitantly stained with titrated amounts of fluorochrome labelled antibodies targeting CD3 APC-H7, CD19 Alexa Fluor 700, CD4 BD Horizon V500, CD8 BD Horizon V450, CD27 PerCP-Cy5.5, CD28 APC, CCR7 PE-Cy7 and CD45RA FITC (all from BD Biosciences) and was incubated for 60 minutes in a light-protected environment at 2°C.

**Figure 1:**
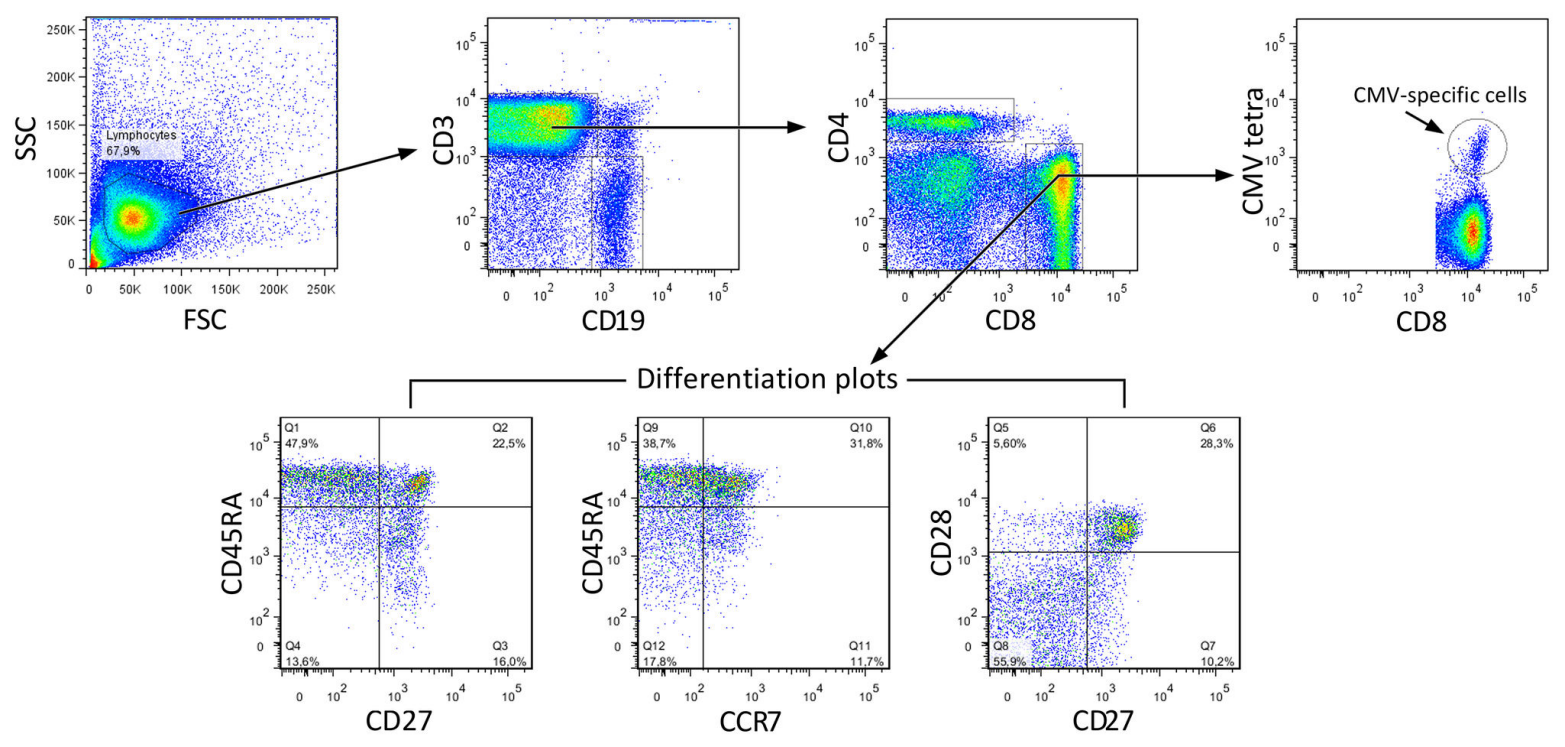
Gating flow-chart. The CD3+CD8+ subset was identified from the FSC/SSC lymphocyte window, and further analysed for CMV tetramer staining and CD27/CD28/CD45RA/CCR differentiation.

All samples were analysed using a BD LSR II Special Order System, controlled by the BD FACSDiva 6.0 software (BD Biosciences). Compensation for spectral overlap was calculated based on data from unstained and single-colour stained BD CompBeads, using the antibody-fluorochrome conjugates specified above. A preliminary forward scatter (FSC) vs. side scatter (SSC) gate was used to identify lymphocytes and, depending on sample size, a total of up to 100 000 in-gate events were recorded. All datasets were migrated to FlowJo 7.6.5 (Treestar Inc.) for further gating and analysis. Gating was performed as specified in Figure 1 and only visually distinct tetramer-positive populations were counted.

### Statistical analysis

R version 2.15.1 (The R Foundation for Statistical Computing) was used for statistical analysis. All comparisons were made using the non-parametric Mann-Whitney U-test. The primary outcome variable was the proportion of CMV-specific cells of total CD8, and ad hoc statistical analyses comparing AD and ND groups were performed without any correction for multiplicity. All other variables were considered secondary and significance tests were adjusted with the Bonferroni correction method. 

Post hoc, to correct for possible influence by age and gender distribution differences between groups, a linear model was constructed correlating the rank of the primary outcome variable with dementia status, age and gender [30]. Box plots were defined with boxes containing quartiles 2 and 3 and whiskers displaying quartiles 1 and 4, excluding any outliers outside 1.5 times the interquartile range.

## Results

### Background data

The epidemiological and laboratory background data of the AD and ND subjects is described in Table 1. As a small number of analytic results were returned blank or erroneous, the mean values have been calculated on the available data.

**Table 1:** Summary of baseline characteristics in patients with Alzheimer’s disease (AD) and non-demented controls (ND).

**Continuous data reported as mean (Standard Deviation**)	**AD (N=50**)	**ND (N=50**)
Age, years	77.5 (6.9)	74.0 (8.0)
Gender, male/female	28/22	22/28
Mini-mental State Examination score	19.9 (4.8)	NA
Haemoglobin, g/L	139.1 (10.6)	137.9 (10.4)
Leukocytes, 10^9^/L	6.75 (1.78)	6.28 (1.67)
Lymphocytes, 10^9^/L	1.95 (0.89)	1.88 (0.61)
C-reactive protein, mg/L	3.59 (7.05)	3.18 (4.84)
Interleukin-6, ng/L	2.24 (2.94)	2.23 (3.08)
*APOE* ε4 allele carriers, hetero-/homozygote	28/4	16/2
CMV IgG positive	84% (n=42)	78% (n=39)
HLA-A02 positive	60% (n=30)	70% (n=36)
HLA-A02 and CMV IgG positive	52% (n=26)	60% (n=30)
HLA-A01/A02/A24/B07/B08/B35 positive	94% (n=47)	90% (n=45)
HLA-A01/A02/A24/B07/B08/B35 positive and CMV IgG positive	78% (n=39)	72% (n=36)

NA = Not Available

The AD and ND groups were similar regarding total leukocyte count, total lymphocyte count, C-reactive protein and interleukin-6. The standard deviation of some parameters exceeded the mean in magnitude, suggesting skewed data distribution. CMV serostatus and HLA allele distribution were also comparable between groups, but the AD group contained a larger proportion of *APOE* ε4 allele carriers. Due to incomplete HLA coverage of available tetramers and CMV serostatus, the total number of participants analysable for CMV MHC-1 specificity was 39 in the AD and 36 in the ND group.

### Flow cytometry

**Figure 2a-c:**
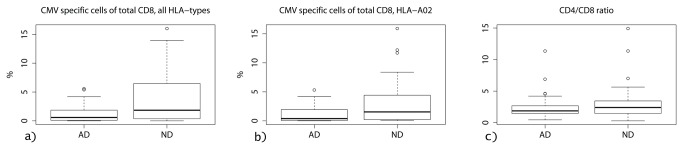
Comparison of proportions of CMV-specific CD8 cells and overall CD4/CD8 ratio in seropositive subjects with Alzheimer´s disease (AD) and non-demented controls (ND). **a**) Comparing total cell count for all HLA-types, there is a clear difference with significantly lower proportions of CMV-specific CD8 cells in AD compared to ND group; 1.16 % versus 4.13 % (p=0.0057) **b**) Comparing only subjects with HLA-A02 tetramer data, there is a trend towards lower proportions of CD8 cells in the AD compared to the ND group; 1.26 % versus 3.07% (p=0.066) **c**) The overall CD4/CD8-ratio did not differ between groups.

The FSC/SSC gate generated an average number of 103 315 events for the AD group and 96 815 for the ND group, respectively. When comparing proportions of the total sum of CMV-specific CD8 cells for all HLA types per CMV positive subject (Figure 2a), there was a significant difference (p=0.0057) between AD (1.16%) and ND (4.13%) groups. This difference was still significant (p=0.0012) when correcting for age and gender. When comparing only HLA-A02 tetramer data from HLA-A02 positive CMV positive subjects (Figure 2b) there was a trend (p=0.066) indicating a similar numerical difference between AD (1.26%) and ND (3.07%) groups, also unaffected (p=0.058) by correction for age and gender. The overall CD4/CD8-ratio did not differ between groups (Figure 2c).

**Figure 3:**
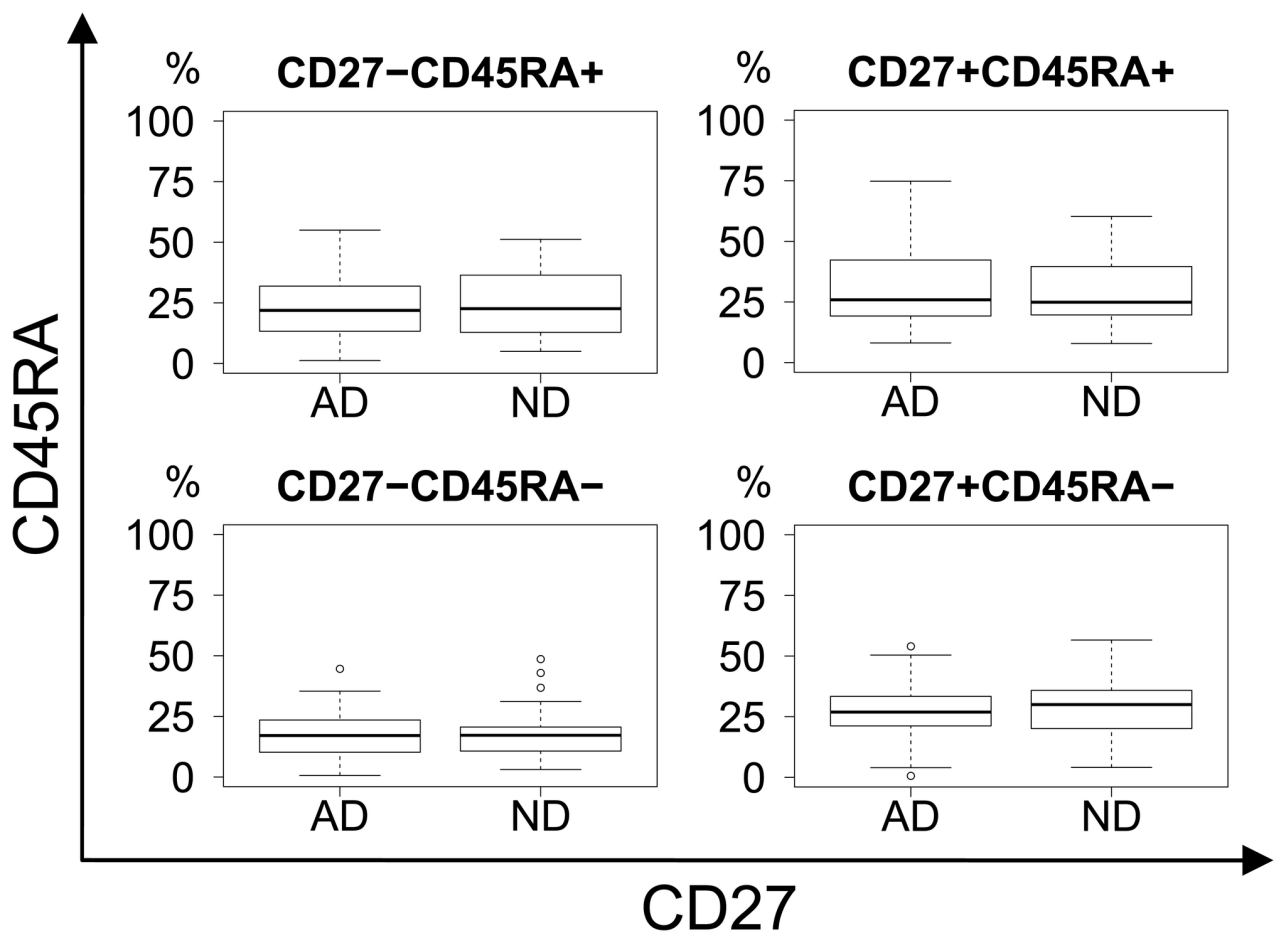
CD27 vs. CD45RA differentiation plot. No difference in differentiation between AD and ND groups in terms of CD27 and CD45RA expression. Upper right: Naïve. Lower right: Memory. Upper left: Effector.

**Figure 4:**
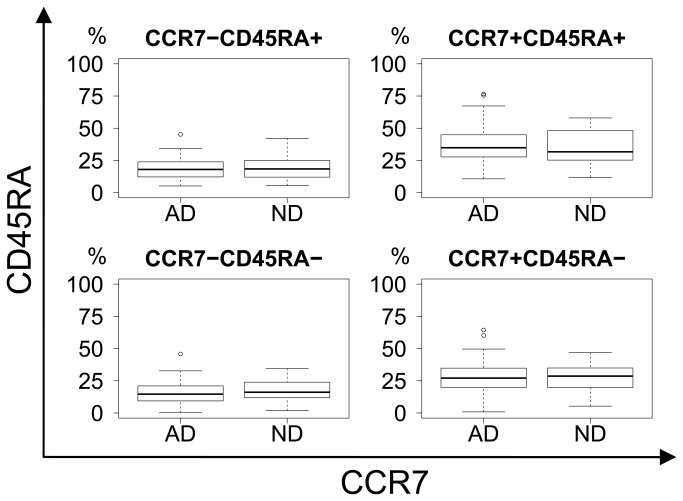
CCR7 vs. CD45RA differentiation plot. No difference in differentiation between AD and ND groups in terms of CCR7 and CD45RA expression. Upper right: Naïve. Lower right: Central memory. Upper left: Effector. Lower left: Effector-memory.

**Figure 5:**
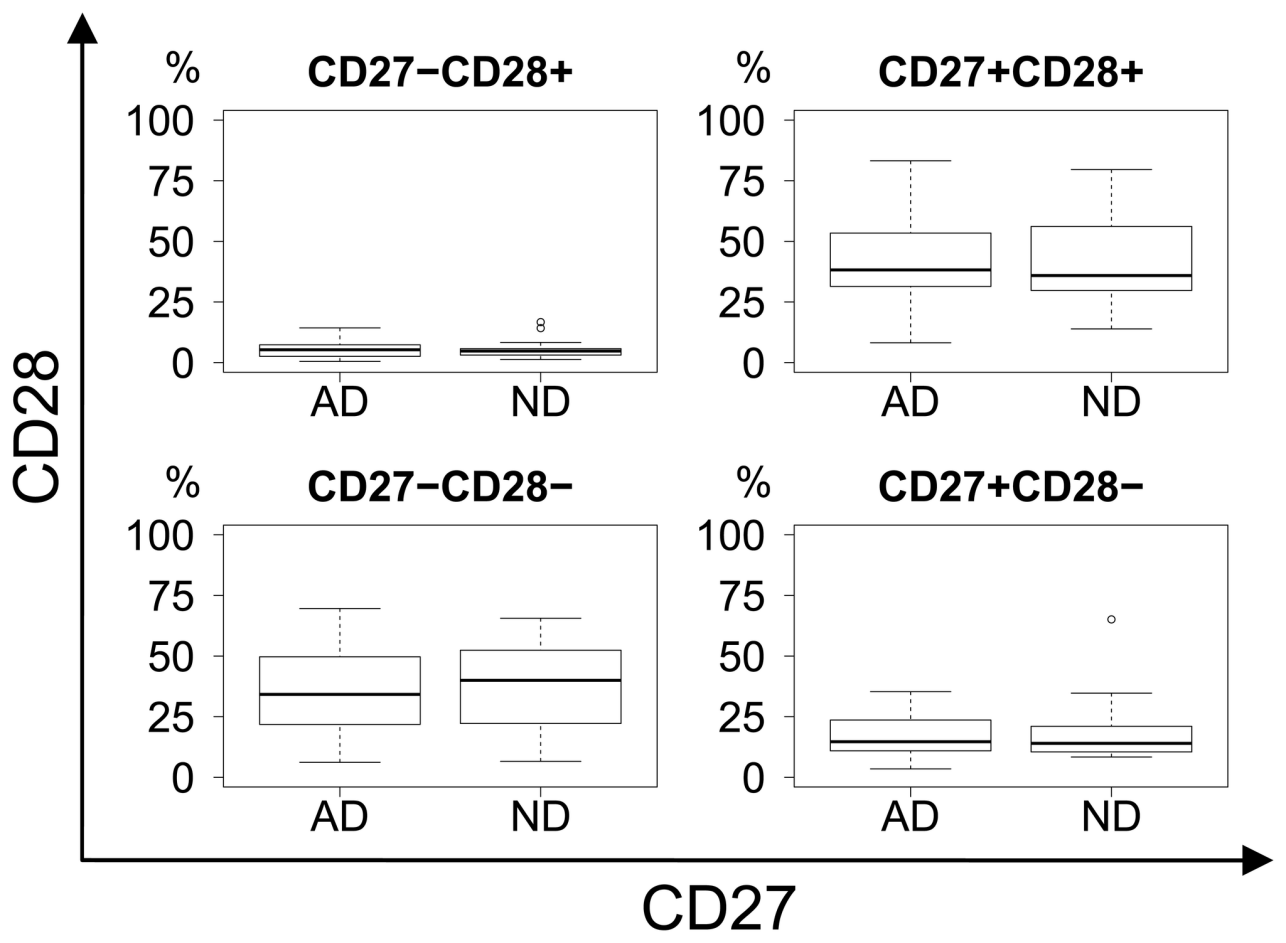
CD27 vs. CD28 differentiation plot. No difference in differentiation between AD and ND groups in terms of CD27 and CD28 expression. Upper right: Early memory and naïve. Lower right: Intermediate memory. Lower left: Late memory.

General CD8 differentiation was illustrated by CD27/CD45RA, CCR7/CD45RA and CD27/CD28-projections of the four-dimensional CD27/CD28/CCR7/CD45RA phenotypic space. No significant differences in differentiation were seen when comparing AD and ND groups (Figures 3-5).

**Figure 6:**
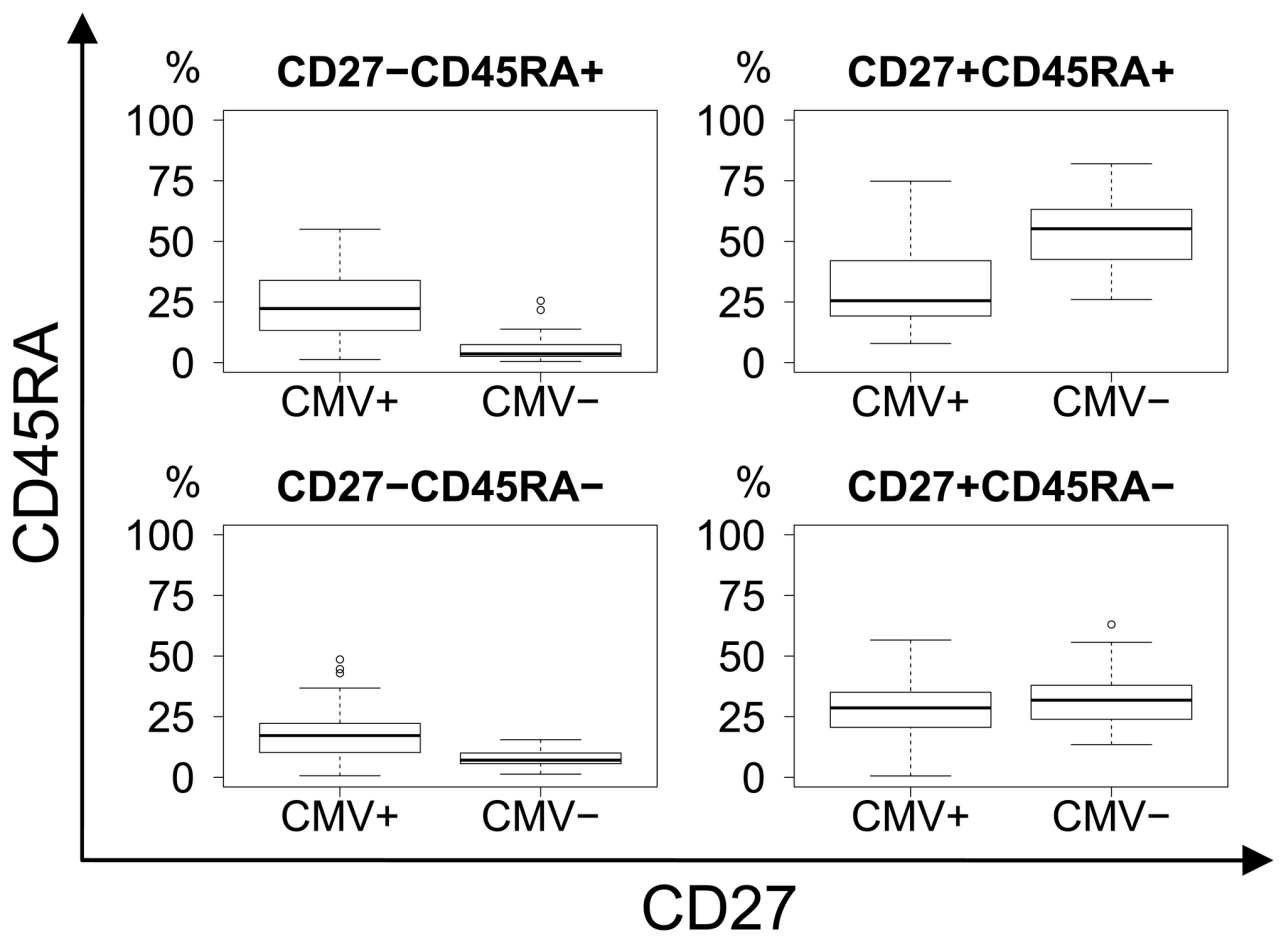
CD27 vs. CD45RA differentiation plot. Significant shift from the CD27+CD45RA+ naïve (p=8.13E-05) to CD27-CD45RA+ effector (p=1.76E-06) and CD27-CD45RA- (p=1.17E-04) subsets with CMV status. Upper right: Naïve. Lower right: Memory. Upper left: Effector.

**Figure 7:**
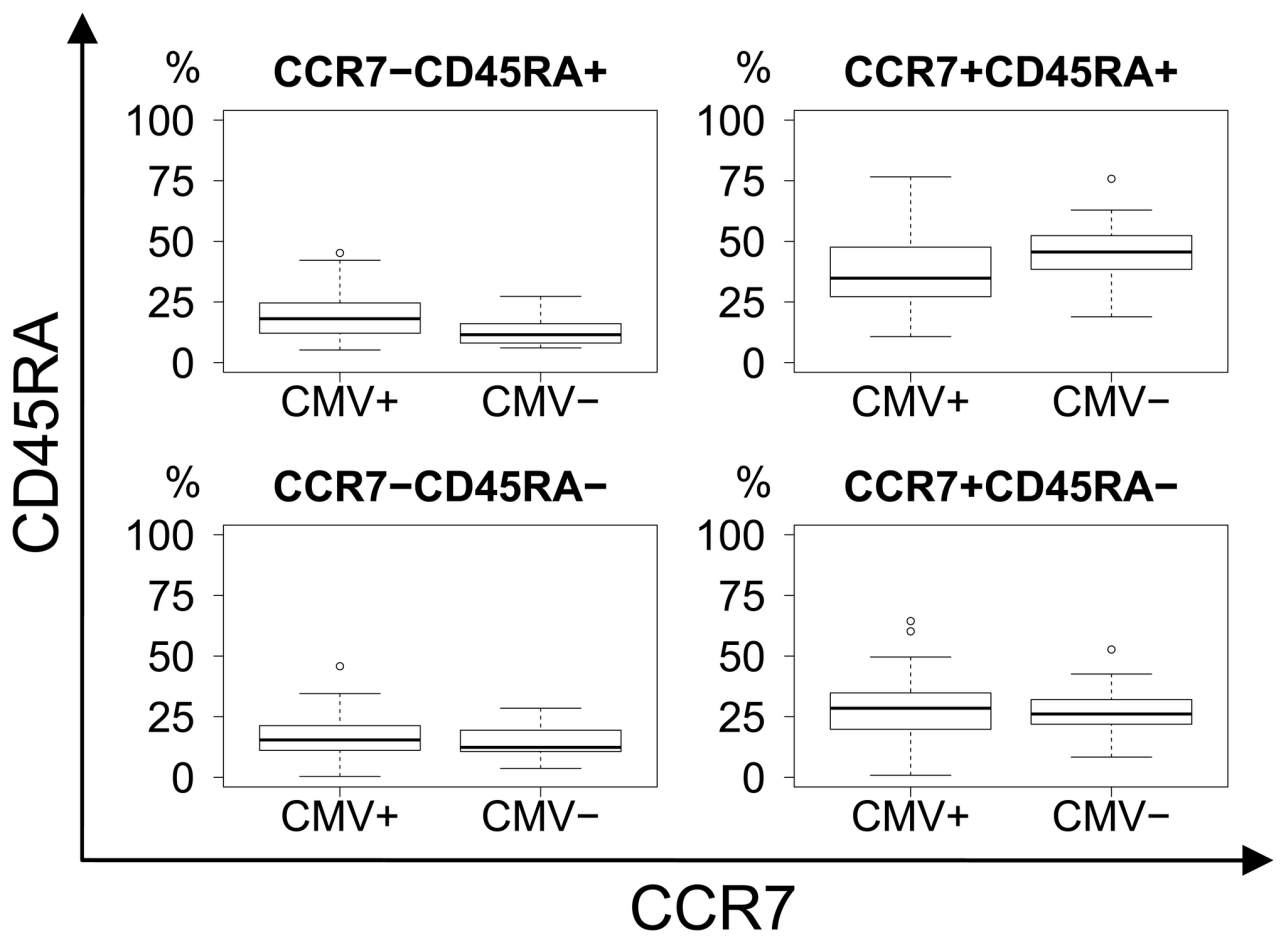
CCR7 vs. CD45RA differentiation plot. Non-significant trend in shift from the CCR7+CD45RA+ naïve (p=0.062) to the CCR7-CD45RA+ effector (p=0.13) subset with CMV status. Upper right: Naïve. Lower right: Central memory. Upper left: Effector. Lower left: Effector-memory.

**Figure 8:**
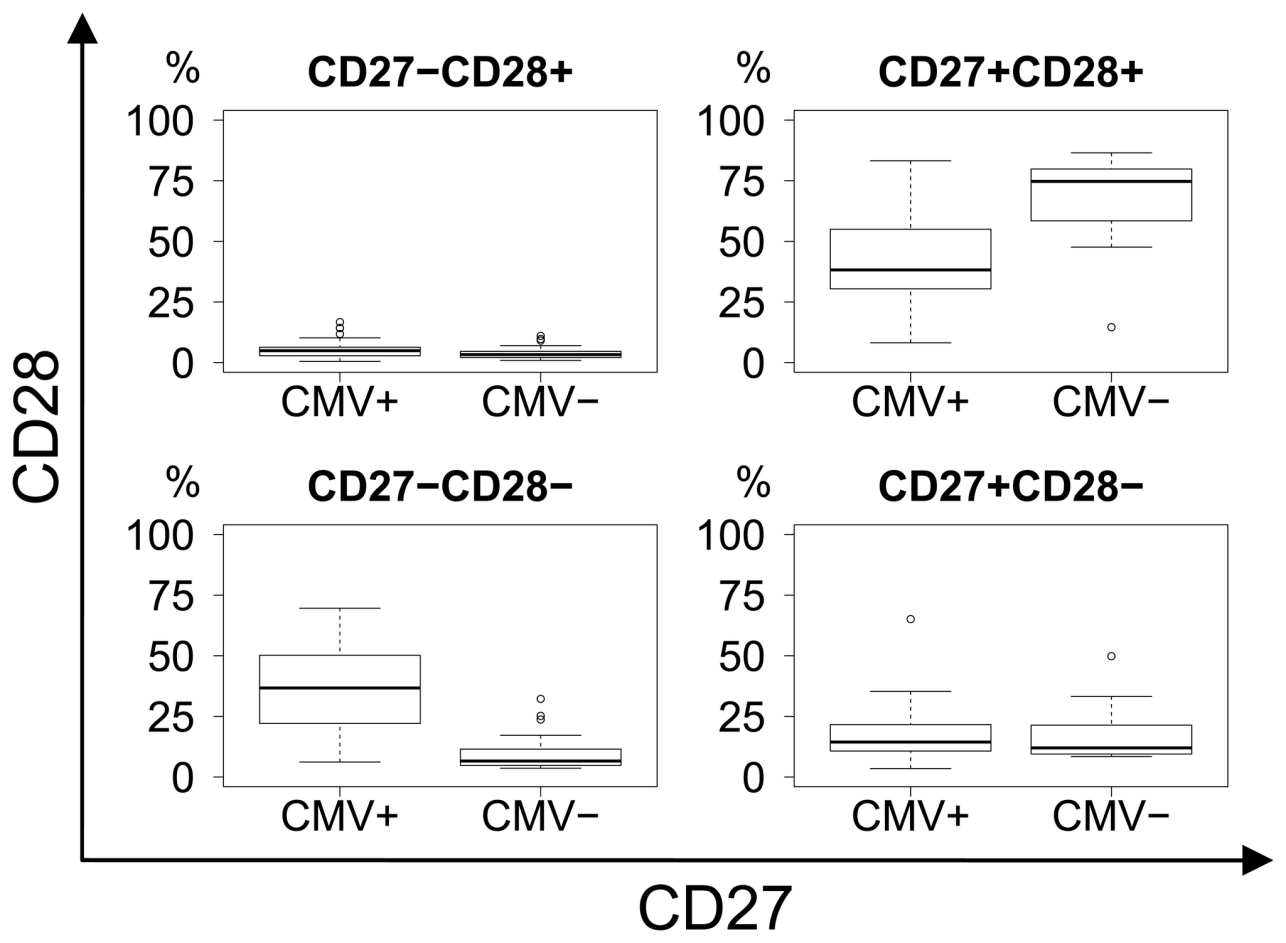
CD27 vs. CD28 differentiation plot. Significant shift from the CD27+CD28+ early memory and naïve (p=3.19E-05) to the CD27-CD28- late memory (p=2.73E-07) subset with CMV status. Upper right: Early memory and naïve. Lower right: Intermediate memory. Lower left: Late memory.

When comparing CMV seropositive and CMV seronegative subjects, regardless of dementia status, there was a clear difference in CD8 differentiation as CMV seropositive subjects presented with substantial shifts in phenotype, from naïve and early memory towards late memory and effector differentiation (Figures 6-8). All p−values were calculated using Bonferroni correction for a multiplicity of 27.

## Discussion

To our knowledge, this is the first study investigating CMV-specific immunity in AD. Studying an age-related disease, we had expected AD patients to present with a phenotype of premature immunosenescence and expanded clones of CMV-specific CD8 cells. Interestingly, this group instead presented with a significantly lower proportion of CMV-specific cells compared to ND controls. There were no differences in denominators such as CD4/CD8 ratio or total lymphocyte count that could explain the difference in CMV specificity, nor any obvious signs of more advanced immunosenescence in the AD group in terms of CD27/CD28/CD45RA/CCR7 differentiation which confirms results from Pellicanò et al [25]. Also, the groups were similar in levels of system inflammatory biomarkers such as C-reactive protein and interleukin-6, providing no evidence that inflammaging is a cofactor for AD development. CMV serostatus and HLA allele distribution were comparable between groups, but as expected [31] the AD group contained a larger proportion of *APOE* ε4 allele carriers.

When comparing CMV seronegative with seropositive subjects regardless of dementia status, significant shifts in the CD8 subsets became obvious. This confirms the results of previous studies that CMV infection itself induces T-cell differentiation towards late effector phenotypes [12,32], but does not infer a link to the risk of developing AD.

If the lower proportion of CMV-specific CD8 cells in AD patients reflects a partially impaired cellular immunity, present before or in early stages of the AD pathophysiological process, CMV reactivation in brain macrophages or vascular endothelial cells could be contributing to local inflammation and disease progression. Another theory would be that CMV immunity, which in normal individuals engages a rather large proportion of the CD8 compartment, is suppressed by AD specific immunological processes, i.e. immunity directed towards amyloid beta or other components related to AD development. 

We believe that our data is relevant, as it is obtained from a fairly large number of AD patients, diagnosed by current clinical protocols and compared to matched controls. We are not yet able to directly link our findings to the AD pathophysiological process but think that they can contribute to new angles of approach to future research. 

There are some inherent methodological weaknesses to this study. Firstly, when working with frozen PBMC samples, there is a risk of losing a proportion of more sensitive cell types, which could possibly skew the results. Efforts to avoid biasing effects include standardised, simultaneous and blinded handling of study and control samples, which should distribute errors evenly between study groups. Secondly, the tetramer staining technique carries limitations, mainly in terms of limited HLA coverage and immune dominance, but as our results are similar when comparing data from multiple HLA types with the subgroup of only HLA-A02 AD/ND subjects, they should be generalisable to other populations. However, the comparison between HLA-A02 AD/ND subjects did not quite reach statistical significance (p=0.058 after correction) which could be due to the HLA-A02 groups being of insufficient size. 

Based on the results of this study we conclude that CMV CD8 T-cell frequency is significantly lower in AD than in non-demented controls, possibly affecting CMV immunity. Any causality between CMV and AD remains to be shown, but we believe that this has brought new insight into how immunity in AD differs from the normal ageing process. Should future studies prove a causative role of CMV in the AD pathophysiology this could increase the benefit of a future CMV vaccine, given that this does not trigger the same pathology as the infection itself. However, we find it more likely that the altered CMV immunity in AD patients reflects more profound changes in systemic immunity and that other forms of immunotherapy might be considered. Further studies in AD, comparing CMV specific CD4 immunity, cellular reactivity on CMV antigen challenge and specific immunity against other chronically persistent viruses, could put this into better context.
